# Infrared Spectroscopy Coupled with Machine Learning Algorithms to Investigate Vascular Dysfunction in Ovariectomy: An Animal Model Study

**DOI:** 10.1021/acsomega.4c08831

**Published:** 2025-01-23

**Authors:** Tháfanys S. Travezani, Márcia H. C. Nascimento, Tagana R. da Cunha, Roger L. dos Santos, Francis L. Martin, Valerio G. Barauna

**Affiliations:** † Department of Physiological Sciences, Federal University of Espirito Santo, Av. Mal. Campos, 1468 - Maruípe, Vitória 29047-105, Espírito Santo, Brazil; ‡ Department of Chemistry, Federal University of Espirito Santo, Av. Fernando Ferrari, 514 - Goiabeiras, Vitória 29075-910, Espírito Santo, Brazil; § Francis L. Martin: Clinical Research Centre, Blackpool Teaching Hospitals NHS Foundation Trust, Blackpool Victoria Hospital, Whinney Heys Road, Blackpool FY3 8NR, U.K.

## Abstract

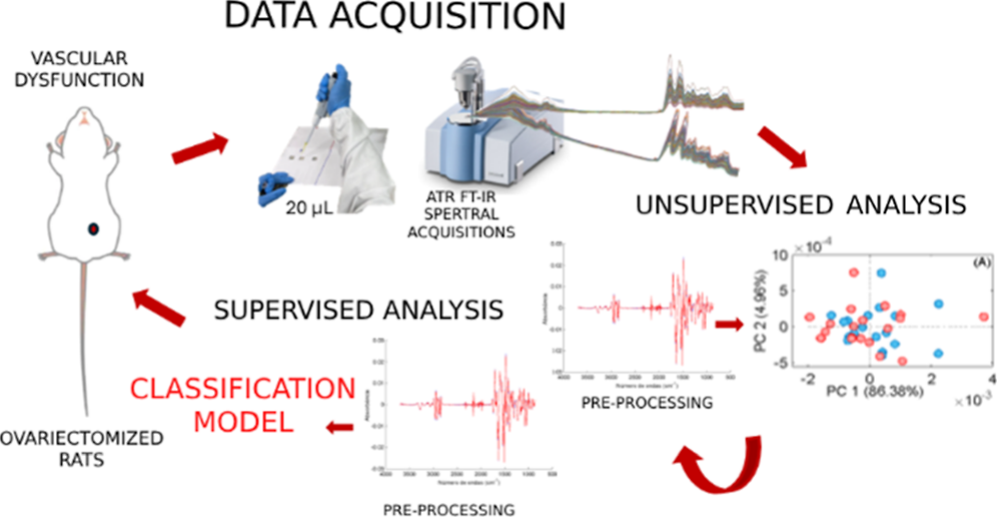

A decrease in female sex hormone levels in the body impairs vascular endothelium functioning, leading to vascular dysfunction associated with certain diseases. Animal models of ovariectomy are commonly used to understand its effects on vascular (dys)function. Fourier-transform infrared (FTIR) spectroscopy is a technique capable of extracting detailed molecular information and, as such, has been applied to various biological analyses. This study evaluated systemic changes in the ovariectomy model using mid-infrared spectroscopy. Thirty-eight serum samples from adult Wistar rats were analyzed and divided into 18 in the control group (SHAM) and 20 in the ovariectomized group (OVX). Bilateral ovariectomy was performed, followed by euthanasia of the rats after 15 days. The spectral collection was performed using the Bruker Alpha II equipment (Bruker, Germany), preprocessed, and analyzed using unsupervised analysis methods [principal component analysis (PCA)] and supervised analysis methods [partial least-squares discriminant analysis (PLS-DA)] (MATLAB 2023). For the PCA model, combinations between principal components (PCs) 1 to 4 were performed. Nevertheless, none of the PC combinations allowed a clear distinction between the OVX and SHAM groups. The PLS-DA model exhibited 66% sensitivity, 80% specificity, a false positive rate of 20%, and a false negative rate of 33%. The F-score was 0.727 and the accuracy was 72.7%. However, the *y*-permutation test demonstrated that this result was random. These results indicate that there is no significant difference in the systemic profile of rats subjected to ovariectomy surgery for 15 days when analyzed using mid-infrared spectroscopy.

## Introduction

Vascular dysfunction is associated with atherosclerotic, cardiovascular diseases and metabolic syndromes.^1−3^ Changes in the vascular bed, which is responsible for producing and releasing vasoactive factors, such as nitric oxide and the endothelium-dependent hyperpolarization (EDH), which regulate smooth muscle tone,^4,5^ are influenced by female sex hormones. The decrease in these hormones in the body can impair the functioning of the vascular endothelium, leading to vascular dysfunction.^5,6^ Therefore, several studies use ovariectomy animal models to understand its effects on vascular dysfunction. However, most focus on discussing local effects, such as in the coronary arteries^7^ or aorta,^8^ while the systemic effects of ovariectomy are only slightly comprehensive.

Fourier-transform infrared (FTIR) spectroscopy with attenuated total reflection (ATR-FTIR) is an analytical technique that, combined with chemometric analyses, has shown great potential in investigating biological samples. The application of this technique allows for the analysis of information at the molecular level, identifying functional groups and providing information about the sample composition.^9^ For the analysis of biological samples, the ATR-FTIR technique has two spectral regions of particular interest: the “fingerprint” region (600–1800 cm^–1^) and the “high wavenumber” region, covering the region rich in lipids and phospholipids (2800–3600 cm^–1^) and, known to contain a variety of biological components such as nucleic acids, proteins, carbohydrates, amino acids, and lipids.^10^ Moreover, ATR-FTIR spectroscopy is a simple approach that requires minimal sample preparation and is easily implementable, in addition to being a nondestructive technique and highly sensitive.^9,11,12^

FTIR technique can be applied to investigate different biological situations. We have used the ATR-FTIR technique as a noninvasive diagnosis tool for SARS-CoV-2 virus infection in saliva and oropharyngeal swabs.^13−15^ Our group also used this technique for patient screening based on the D-dimer^16^ value and to analyze the plasma of mice with sepsis^17^ and iron overload.^18^ Previous studies have used ATR-FTIR to analyze molecular differences in ovariectomy animal models, measuring biochemical variations in tissues based on the vibrational signature of their components. For instance, FTIR was used to compare biochemical changes in the aorta of control and ovariectomized groups. The results demonstrated alterations in the distribution and composition of lipids and proteins in the structure of the aorta, indicating changes in the biochemical properties of vascular tissue in response to ovarian loss.^19^ These studies highlight the versatility and potential of ATR-FTIR spectroscopy, suggesting its usefulness in characterizing vascular alterations.

In the literature, it is possible to find several studies on vascular dysfunction in ovariectomized animal models.^20−22^ However, in a model with 15 days of ovariectomy, the systemic molecular alterations associated with this condition are still poorly understood. In this short interval, circulating hormone levels are already reduced, impacting the body’s homeostasis and causing vascular dysfunction. Thus, understanding the systemic changes related to vascular dysfunction briefly after ovariectomy is essential to elucidate the initial effects of hormonal depletion on the vascular system. We speculate that FTIR spectroscopy can capture the vibrational fingerprint of circulating biomolecules, providing a unique and sensitive signature to the ovariectomy and control group. To this end, machine learning algorithms were applied to analyze complex spectral data and develop models that accurately classify samples. Therefore, this study aimed to investigate systemic changes in an animal model of vascular dysfunction ovariectomy-induced using ATR-FTIR spectroscopy. Chemometric methods such as principal component analysis (PCA) and partial least-squares-discriminant analysis (PLS-DA) were applied to understand the dissimilarity between the groups.

## Methods

### Study Design

In this study, thirty-eight female 8-week-old adult Wistar rats (*Rattus norvegicus*) were raised at the animal facility in the Centre of Health Sciences at UFES. The Institutional Ethics Committee approved all Animal Care and Use procedures of the Federal University of Espirito Santo under protocol # 063/2017. The animals were housed in collective cages (up to five animals per cage) and received food (Purina Labina) and water ad libitum. They were kept under controlled temperature conditions (22–24 °C) and humidity (40–60%), with a 12/12 h light–dark cycle. The animals were randomly assigned to one of two groups: the ovariectomy group (OVX), in which the ovaries were surgically removed, and the sham group (SHAM), which underwent a simulated ovariectomy procedure without removal of the ovaries.

### Ovariectomy

Ovariectomy was induced as previously described by our group^23,24^ and others.^25^ Ovariectomy was performed under general anesthesia with ketamine (80 mg/kg) and xylazine (12 mg/kg), i.p. The rats were subjected to a bilateral incision in the skin followed by an incision in the muscular layer, opening the peritoneal cavity for posterior ligation of the uterine horn and removal of the ovaries. The tube was ligated with a suture line and the ovaries were removed. Muscle and skin were then sutured. After the surgery, the animals received an antibiotic injection (2.5% enrofloxacin, 0.1 mL, i.m.). SHAM rats were incised and sutured, but the ovaries were left intact.^26^

### Serum Collection

Fifteen days after ovariectomy, the animals were euthanized by decapitation, and 5 mL of blood samples were immediately collected. Subsequently, the blood was centrifuged (Excels IV model, model 280r) at 3500 G for 15 min at 4 °C, and the resulting serum was extracted and stored at –20 °C for subsequent analyses. The samples were divided into two distinct groups: 20 serum samples from rats subjected to bilateral ovariectomy (OVX) and 18 samples from animals subjected only to surgery simulation (SHAM).

### Coronary Perfusion Pressure Measurement

The Langendorff perfusion method used in this study has been previously described in detail.^27^ The hearts of animals anesthetized with ketamine (80 mg/kg) and xylazine (12 mg/kg), i.p., were extracted and transferred to the Langendorff apparatus (Hugo Sachs Electronics, March-Hugstetten, Germany). Afterward, the aorta was cannulated and perfused with modified Krebs solution containing 120 mM NaCl, 1.25 mM CaCl_2_·2H_2_O, 5.4 mM KCl, 2.5 mM MgSO_4_ 0.7H_2_O, 2.0 mM NaH_2_PO_4_·H_2_O, 27.0 mM NaHCO_3_, 1.2 mM Na_2_SO_4_, 0.03 mM EDTA and 11.0 mM glucose, continuously heated at 37 °C in water and equilibrated with a mixture of 95% oxygen and 5% carbon dioxide at a controlled pressure of 100 mmHg to yield a pH of 7.4. Coronary flow was kept constant at 10 mL/min so that changes in Coronary Perfusion Pressure (CPP) would be directly related to changes in vascular resistance. Left ventricular (LV) isovolumetric pressure was maintained by inserting a latex balloon into the LV, which was pressurized to maintain intraventricular diastolic pressure at 10 mmHg. After 40 min of stabilization, the baseline CPP was determined.^26^

### ATR-FTIR Spectroscopy

For spectral collection, the samples were thawed at controlled temperature and humidity for about 30 min. Then, 20 μL of serum of each sample was pipetted onto an aluminum plate wrapped in aluminum foil in triplicate. The plates with the samples were kept at room temperature for a minimum period of 2 h to allow the water in the samples to evaporate, as the high absorption of water in the infrared could interfere with the analysis results (temperature: 19.4% ± 0.81%, humidity: 49.4% ± 8.3%).

The spectra were obtained using the ALPHA II spectrometer (ver. 7.8, Bruker, Germany), with OPUS 8.5 software, in the spectral range of 400 to 4000 cm ^–1^, employing attenuated total reflection (ATR) accessory, in absorbance mode with a resolution of 4 cm ^–1^, and 32 scans. Spectra were acquired in triplicate, and for each analysis, the diamond sampling window and the sample press tip were cleaned with 70% ethanol and dried with absorbent paper.

### ATR-FTIR Chemometric Analysis

Data analysis was conducted using MATLAB 13A. Both models (PCA and PLS-DA) shared a common preprocessing foundation. The region below 900 cm^–1^ was excluded due to baseline shifts. Following this, spectral triplicates were averaged and processed for baseline correction using the Adaptive Iteratively Reweighted Penalized Least Squares (airPLS) algorithm.^28^ Subsequently, a 5-point Savitzky–Golay filter was applied for smoothing, which enhances spectral quality and reduces noise.^29^ The models were constructed using the entire spectral range from 4000 to 900 cm^–1^. While the preprocessing steps were consistent across both models, some differences were specific to each PCA and PLS-DA approach, which will be detailed below.

The PCA model used the 15-point Savitzky–Golay second derivative (second-degree polynomial) and mean-centering the data. PCA aims to identify patterns in the data set through linear combinations of the original variables to capture the most significant variation. PCA is an unsupervised method used to reduce the dimensionality of data while preserving most of its information. Principal components (PCs) are ordered in importance, with the first principal component capturing the most variation, followed by the second, and so on. The original mean-centered spectral data matrix was restructured into a new data set of scores and loadings, decomposed into 10 PCs. This dimensional representation in scores was obtained by calculating the dot product between the original and loading data vectors.

The PLS-DA was conducted using a supervised method that linearly reduces data dimensionality by combining predictors to generate latent variables (LVs), forming a new mean-centered dimensional space. This analysis included the application of multiplicative scatter correction (MSC) and second derivative (21 points). The PLS-DA algorithm can generate predictive and descriptive models based on the linear relationship between the matrix of variables and the categorical classes, transforming real variables into new latent variables. The first latent variable of PLS-DA is responsible for preserving the maximum possible covariance among the original samples. In constructing the classification model, 70% of the samples were selected for training and 30% for testing, using the Kennard Stone sampling, maintaining the existing proportion of samples in each class.^30^ The model was validated through sensitivity, specificity, accuracy, false positive rate, false negative rate, and F-score tests.

### Statistical Analysis

Statistical tests were conducted using GraphPad Prism 9.0 software. The Mann–Whitney test was used for all comparisons between groups. Statistical significance was established at *p* < 0.05, and the results were expressed as mean ± standard deviation.

## Results and Discussion

Body weight increase and uteri weight decrease are remarkable characteristics of ovariectomized rats.^31^ Our model of 15 days of ovariectomy exhibited 19% higher body weight (SHAM: 216.0 ± 15.8 vs OVX: 258.4 ± 24.3, grams, *p* < 0.05) than the SHAM animals. Uterine weight in the OVX group was notably 62% smaller than those in the SHAM group (SHAM: 0.31 ± 0.09 vs OVX: 0.12 ± 0.04, grams, *p* < 0.05), indicating the absence of hormones typically produced by the ovaries. To confirm the vascular dysfunction in our model, baseline CPP was measured, and it was lower in the OVX group compared to the SHAM group (SHAM: 92.1 ± 10.6 mmHg vs OVX: 66.7 ± 5.4 mmHg, *p* < 0.05). Taken together, the reduction in uterine weight and baseline CPP confirm the successful ovariectomy in the animal model as previously published by our group.^23,24,26^

### Spectral Analysis

**Figure 1:**
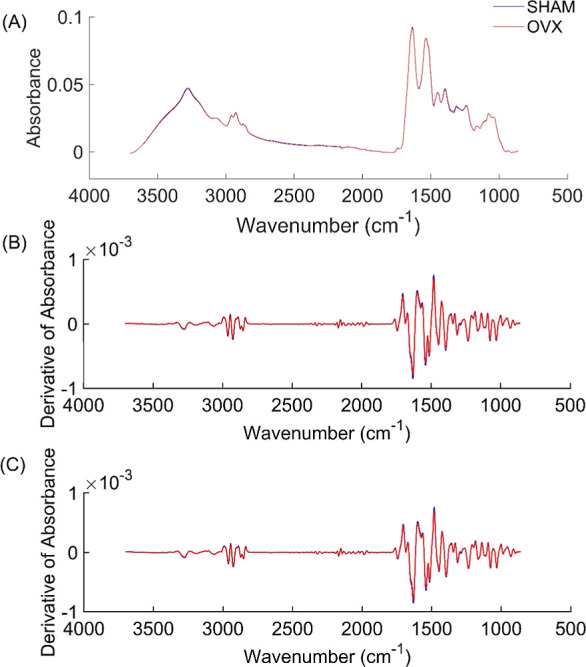
(A) Average FTIR spectra of each group; (B) preprocess method chosen for the PCA: baseline correction, smoothing and second derivative (C) preprocess method chosen for the PLS-DA: baseline correction, smoothing, second derivative and MSC. Sham group is represented by the blue line and the ovariectomized group by the red line.

FTIR spectra were collected in the serum for each group (OVX and SHAM) to study the systemic differences in the chemical compositions of the blood. Figure 1A shows the FTIR average spectra of serum samples from both groups. Various preprocessing methods were tested for building the PCA and PLS-DA models (see Supporting Information 1), with the best-performing one selected for each model. Thus, the processing method utilized to perform the unsupervised model (PCA) is displayed in Figure 1B, while Figure 1C shows the spectra processing method used for the supervised model (PLS-DA).

**Figure 2:**
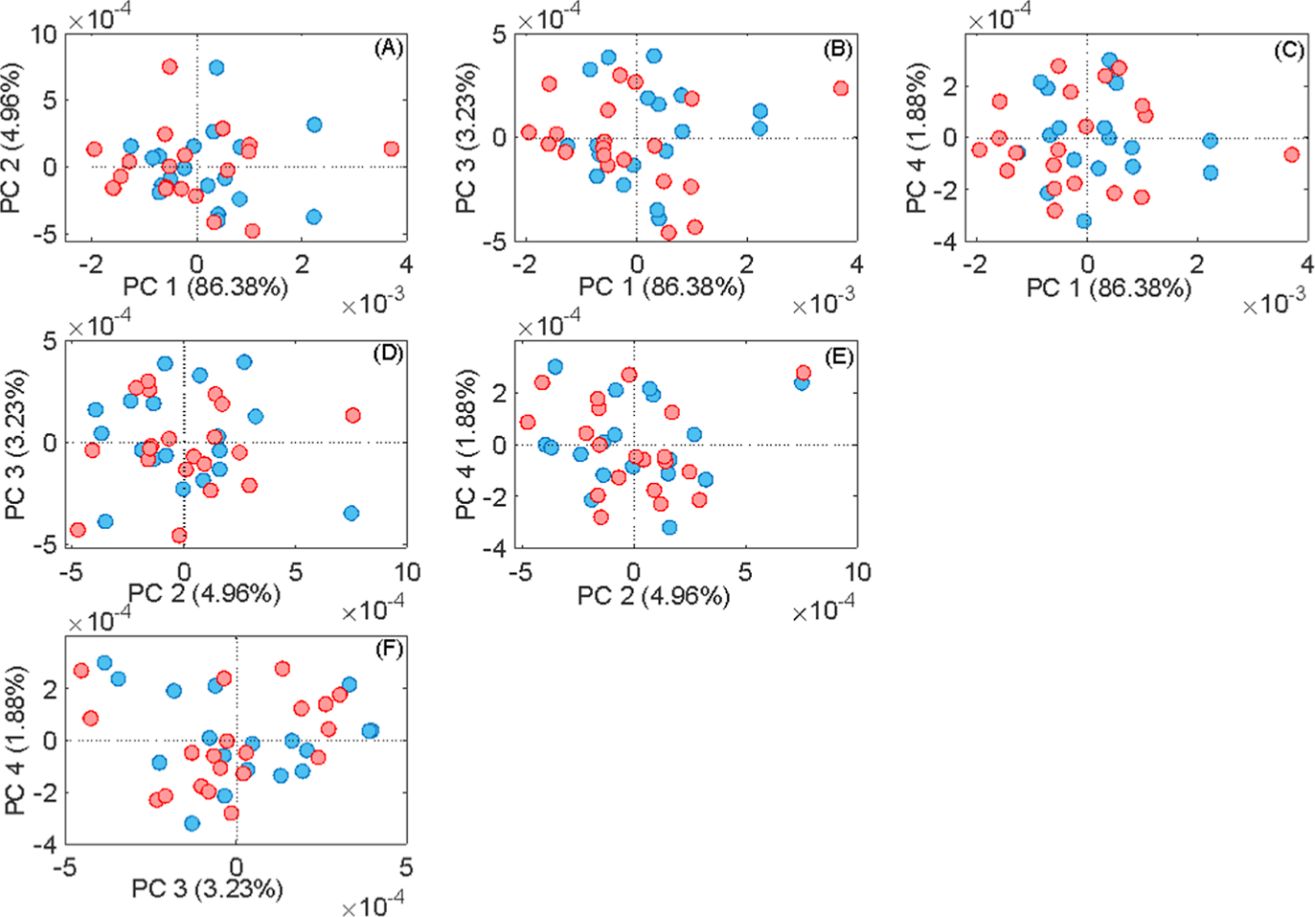
PCA scores plot of PC1 vs PC2, PC3, and PC4; PC2 vs PC3 and PC4; and PC3 vs PC4 in the total spectra. The SHAM group is in blue, and the OVX group is in red.

The PCA approach was utilized to reduce the dimensionality of the FTIR spectral data, enabling visualization through a linear transformation onto a new space composed of orthogonal vectors. The correlation matrix eigenvectors from the set of spectra provide the foundation for the derived matrix transformations. A score value along each principal component represents each sample in the new space. Therefore, we applied PCA as an unsupervised method to visualize differences between the serum of the SHAM and OVX groups using FTIR spectroscopy data. Figure 2 shows the scatter plots of PCA, displaying combinations of PCs (PC1 to PC4) to illustrate the data variance better. PC1 vs PC2 accounted for 91.34% of the explained variance, PC1 vs PC3 for 89.61%, PC1 vs PC4 for 88.26%, PC2 vs PC3 for 8.19%, PC2 vs PC4 for 6.84%, and PC4 vs PC5 for 5.11%. However, none of these PC combinations allowed for the visual distinction between groups.

We analyzed PCA scores to determine whether significant statistical differences exist in two-dimensional space despite the lack of visual separation between groups. None of the first four PCs showed statistically significant differences (PC1, *p* = 0,37; PC2, *p* = 0,98; PC3, 0,59; PC4, *p* = 0,69). This result can be justified since, although PCA is a powerful method for analyzing chemical differences in spectral measurements, it will only capture differences between measurements in their scores if these differences are the main factors contributing to the total variability. Furthermore, intra- and interindividual variability can make it challenging to obtain a clear separation between samples.^32,33^ Thus, other sources of variation that are not representative of the condition of interest can overshadow the biomolecular information in the samples, such as lipids, proteins, carbohydrates, and nucleic acids, which are characteristic of the condition studied.^34^

In this way, the results presented by the PCA model can be explained since variations in correlations can be attributed to the complex metabolic and physiological changes induced by ovariectomy, which can affect serum concentrations of biochemical parameters. Thus, it affects the spectral characteristics of serum samples and specific individual variability in the body’s response and adaptation to the procedure, which can cause a breakdown in the relationships between the spectral characteristics and the evaluated parameters.^32,33,35,36^

Next, the PLS-DA model was performed. Similar to PCA, PLS-DA linearly reduces the data dimensionality. However, it can be done by combining predictors to generate latent variables (LVs). In contrast to PCA, which operates without prior knowledge of the outcome, PLS-DA is a supervised method. This means that in PLS-DA, the analyst needs to provide explicit response values for each measurement, which will guide the model in learning the relationship between the predictors and the response variable.

Therefore, the algorithm was trained using 70% of the samples, while the remaining 30% served as independent test samples. Table 1 presents the performance of the model evaluated through the sensitivity calculation. The training set reached 100% sensitivity, while in the test set it was 66%. As for specificity, the training set achieved 100%, and the test set achieved 80%. For the false positive rate, the training set recorded 0%, while in the testing set it was 20%. On the other hand, the false negative rate was 0% in the training set and 33% in the test set. Lastly, the accuracy was 100% on the training set and 72.7% on the test set.

**Table 1:** Performance Measures and Characteristics of the PLS-DA Model of 38 Serum Samples from Ovariectomized and SHAM Rats Evidenced by ATR-FTIR^a^

set	LV	SENS (%)	SPEC (%)	FPR (%)	FNR (%)	F-score	ACC
training	4	100	100	0	0	1,00	100
test	4	66.7	80.0	20.0	33.3	0.727	72.7

a LV: Latent variables; SENS: Sensitivity; SPEC: specificity; FPR: False Positive Rate; FNR: False Negative Rate; ACC: Accuracy.

**Figure 3:**
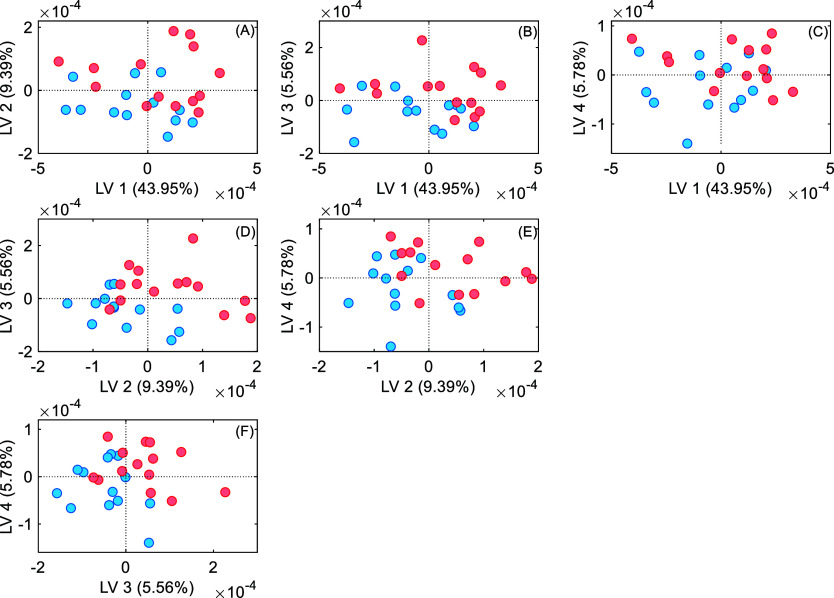
PLS-DA scores plots of LV1 vs LV2, LV3, and LV4; LV2 vs LV3 and LV4; and LV3 vs LV4 in the total spectra. The SHAM group is in blue, and the OVX group is in red.

The scatterplot with the resulting latent variables (LVs), obtained through the linear reduction of the dimensionality of the spectral data, is illustrated in Figure 3. The covariances between samples from both groups (LVs 1 to 5) are presented. Thus, LV1 vs LV2 presented 53.34% of covariance between samples, LV1 vs LV3 49.51%, LV1 vs LV4 49.73%, LV2 vs LV3 14.95%, LV2 vs LV4 15.17% and LV4 vs LV5 11.34%. Like PCA, the PLS-DA model did not clearly separate the groups despite the model’s training of good specificity and sensitivity.

**Figure 4:**
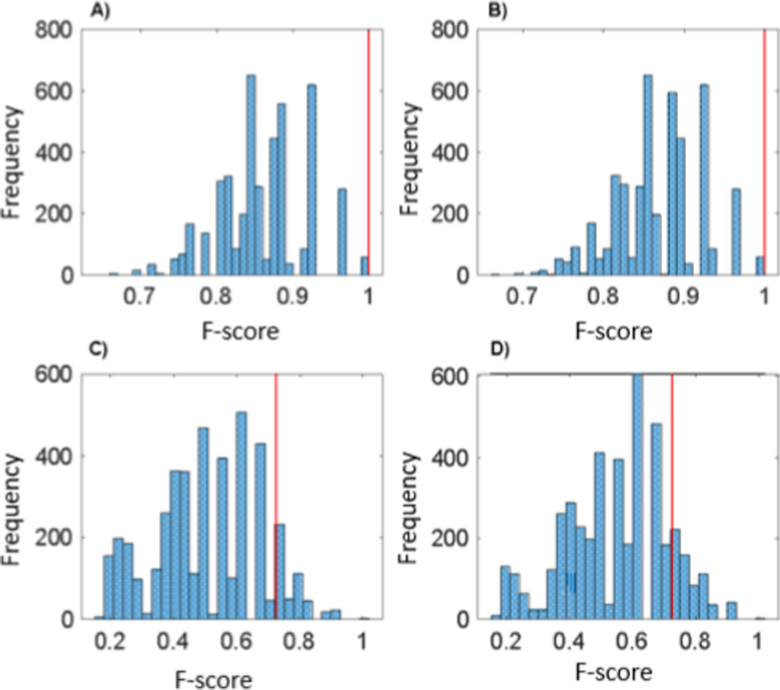
Graphical visualization of the *y*-permutation test: F-score histogram of permuted PLS-DA models vs F-score of the original model (red vertical line), Full Spectrum (3700–900 cm^–1^). (A) Training set, SHAM class. (B) Training set, OVX class. (C) Test set, SHAM class. (D) Test set, OVX class.

Figure 4 displays the *y*-permutation test graph, illustrating models created with permuted sample classes to disrupt cause-effect relationships. Models using randomized data show a performance distribution in a histogram alongside a vertical line representing the model’s performance with correct class assignments. This test evaluates whether associations between independent variables and the dependent variable are real or random. In the training set, OVX and SHAM groups achieved an F-score of 1 without randomness, indicating clear group distinction. Models with randomized classes mostly scored between 0.7 and 0.9 in training, approaching those with real classes. In the test set, both groups scored 0.7 with real classes, indicating difficulty in distinguishing SHAM from OVX in new data. While most randomized models scored below 0.7 in the test set, some scored between 0.7 and 1.0, integrating this study’s model performance into the distribution. However, *y*-permutation testing in binary classification may yield misleading results, as some permuted models retain correct labels, influencing pseudorandom outcomes. Therefore, evaluating additional factors alongside *y*-permutation testing, such as key variables, is crucial to assess the analysis’s chemical significance.

The metrics presented by the model in the training set were satisfactory; however, the same performance was not achieved in the test set, which offered lower metrics than the training set. Furthermore, the permutation test applied to identify the randomization of the model demonstrated that the result presented was not real.

In the current literature, there are no studies that conduct systemic investigations using the ATR-FTIR spectroscopy technique in ovariectomized animal models; all studies found using the method are focused only on local investigations, such as changes in bone composition or vascular changes in the aortic tissue of animals after ovariectomy. Furthermore, these studies that use the ATR-FTIR spectroscopy technique for local analyses involve a long-term ovariectomy, unlike the model we used, which was for a short period of 15 days.^19,37−39^

In our literature review, we also did not find many studies using 15-day ovariectomy models, with the vast majority of studies focusing on local and not systemic analyses. Our study demonstrated that 15 days of ovariectomy caused local changes as an increase in baseline CPP, which may be related to vascular dysfunction. As well as other nonsystemic studies in rats ovariectomized for 15 days also detected local changes in the vascular endothelium, such as increased oxidative stress and reduced bioavailability of nitric oxide (NO), both leading to increased vascular resistance. They also detected changes in the vasodilatory response of the coronary artery and endothelial disorders that may indicate vascular dysfunction.^23,24,26^ In addition to these changes related to vascular dysfunction, another study carried out over 7 days demonstrated that ovariectomy caused changes in the preoptic area of the hypothalamus, showing smaller and different activities during temperature stimuli after ovariectomy.^40^ Another study using rats ovariectomized for 18 days demonstrated that the antioxidant status in cardiac tissue and erythrocytes were seriously compromised by OVX and detected changes in plasma values of creatine kinase (CK), aspartate aminotransferase (AST) and alanine aminotransferase (ALT), which may be associated with cellular damage.^41^

Studies of 15 days or less of ovariectomy investigating systemic changes were carried out using metabolomic methods.^42,43^ These methods have a more specific investigation capacity compared to ATR-FTIR, which performs a general analysis of the molecular information present in the samples. Another systemic study was conducted, which analyzed arterial blood to investigate bleeding disorders.^44^ Given this information, it is indicative that the 15-day period used in our model may have been a determining factor for the nondistinction between the SHAM and OVX groups. Another possibility is that the ATR-FTIR technique, together with the PCA and PLS-DA models, was not able to capture changes in the serum of ovariectomized animals, as the ovariectomy time may not have been sufficient to generate significant changes detectable by this method.

## Conclusion

This study applied ATR-FTIR spectroscopy combined with chemometric analyses utilizing unsupervised (PCA) or supervised (PLS-DA) models to determine if it could distinguish serum from animals with probable vascular reactivity dysfunction. However, neither model could clearly separate the samples between the control and vascular reactivity dysfunction groups despite the PLS-DA model showing reasonable specificity and sensitivity. Given the well-known capability of ATR-FTIR spectroscopy combined with multivariate analyses in extracting relevant information from biofluids, we believe that the 15-day duration of the model may have limited the detection of biochemical changes, thus influencing the overall differentiation between the SHAM and OVX groups.

## Supporting Information Available

Supporting Information to the methods section Raw Spectral Data Set (XLSX)
Figure S1. PCA scores plot from the total spectra were preprocessed with multiplicative scatter correction. Figure S2. PCA scores plot from the total spectra, preprocessed with Multiplicative Scatter Correction and second derivative (15 points). Figure S3. PCA scores plot from the total spectra, preprocessed with Multiplicative Scatter Correction and second derivative (21 points). Figure S4. PCA scores plot from the total spectra, preprocessed with normalization. Figure S5. PCA scores plot from the total spectra, preprocessed with normalization and second derivative (21 points). Figure S4. PCA scores plot from the total spectra, preprocessed with Standard Normal Variate and second derivative (21 points). Table S1. Performance measures and characteristics of the PLS-DA model preprocessed with Multiplicative Scatter Correction. Table S2. Performance measures and characteristics of the PLS-DA model preprocessed with Multiplicative Scatter Correction and second derivative (19 points). Table S3. Performance measures and characteristics of the PLS-DA model preprocessed with normalization. Table S4. Performance measures and characteristics of the PLS-DA model, preprocessed with second derivative (19 points). Table S5. Performance measures and characteristics of the PLS-DA model preprocessed with normalization and second derivative (PDF)
