# Supplementary material for: Infrared Spectroscopy Coupled with Machine Learning Algorithms to Investigate Vascular Dysfunction in Ovariectomy: An Animal Model Study
Source: ACS Omega. 2025 Jan 23;10(4):3701–8. doi: 10.1021/acsomega.4c08831 (PMC11800005; doi:10.1021/acsomega.4c08831)
Supplement: Supplementary file 2 — ao4c08831_si_002.pdf [file ao4c08831_si_002.pdf]

Supporting Information

Infrared spectroscopy coupled with machine learning algorithms to investigate vascular dysfunction in ovariectomy: an animal model study

Tháfanys S. Travezani[a], Márcia H. C. Nascimento[b], Tagana R. da Cunha[a], Roger L. dos Santos[a], Francis L. Martin[c], Valerio G. Barauna[a]

[a]Department of Physiological Sciences, Federal University of Espirito Santo, Av. Mal. Campos, 1468 - Maruípe, 29047-105, Vitória, ES, Brazil.

[b]Department of Chemistry, Federal University of Espirito Santo, Av. Fernando Ferrari, 514 - Goiabeiras, 29075-910, Vitória, ES, Brazil.

[c]Francis L. Martin: Clinical Research Centre, Blackpool Teaching Hospitals NHS Foundation Trust, Blackpool Victoria Hospital, Whinney Heys Road, Blackpool FY3 8NR, U.K.

e-mail: *Travezani.t.s@gmail.com

Supporting Information

Summary

1- Supporting information to the methods section
2- Raw Spectral Data Set (excel file)
3- **Figure S1**. PCA scores plot from the total spectra were pre-processed with multiplicative scatter correction.
4- **Figure S2.** PCA scores plot from the total spectra, pre-processed with Multiplicative Scatter Correction and second derivative (15 points).
5- **Figure S3.** PCA scores plot from the total spectra, pre-processed with Multiplicative Scatter Correction and second derivative (21 points).
6- **Figure S4.** PCA scores plot from the total spectra, pre-processed with normalization.
7- **Figure S5.** PCA scores plot from the total spectra, pre-processed with normalization and second derivative (21 points).
8- **Figure S4.** PCA scores plot from the total spectra, pre-processed with Standard Normal Variate and second derivative (21 points).
9- **Table S1.** Performance measures and characteristics of the PLS-DA model pre-processed with Multiplicative Scatter Correction.
10- **Table S2.** Performance measures and characteristics of the PLS-DA model pre-processed with Multiplicative Scatter Correction and second derivative (19 points).
11- **Table S3.** Performance measures and characteristics of the PLS-DA model *p*re-processed with normalization.
12- **Table S4.** Performance measures and characteristics of the PLS-DA model, *p*re-processed with second derivative (19 points).
13- **Table S5.** Performance measures and characteristics of the PLS-DA model *p*re-processed with normalization and second derivative.
14- **References**

Supporting information about the methods section

Unsupervised analysis

Principal Component Analysis (PCA) is an unsupervised method widely used for initial exploratory analysis of infrared spectroscopy data. It allows for identifying patterns and relationships between variables, clusters, and anomalous samples without the need for class labeling. The method performs a linear reduction of data dimensionality through principal components (PCs), retaining the most relevant information. In this process, the initial matrix (X_{ij}) is decomposed into a score matrix (T_{ih}), which represents the samples in the new coordinates, and a loading matrix (P_{hj}), which indicates the contribution of each variable to the PCs. The principal components are ordered based on the amount of information they retain, with the first principal component (PC1) capturing the largest portion of information, as it is derived from the first decomposition of the *X* matrix, followed by the second, and so on[1,2].

Supervised analysis

Partial Least Squares Discriminant Analysis (PLS-DA) is a supervised classification method that performs linear dimensionality reduction of the data through latent variables (LV) to capture relevant information present in the data matrix *X*, combined with the response vector y. In this method, the vector *y* contains class information, allowing the determination of which class an unknown sample belongs to based on the provided data. For each sample, 1 is assigned to the class of interest and 0 to the other classes. The probability of belonging to each class is calculated, and the sample is classified into the class with the highest probability[3,4].

Raw Spectral Data Set

Excel file attached

PCA MODELS

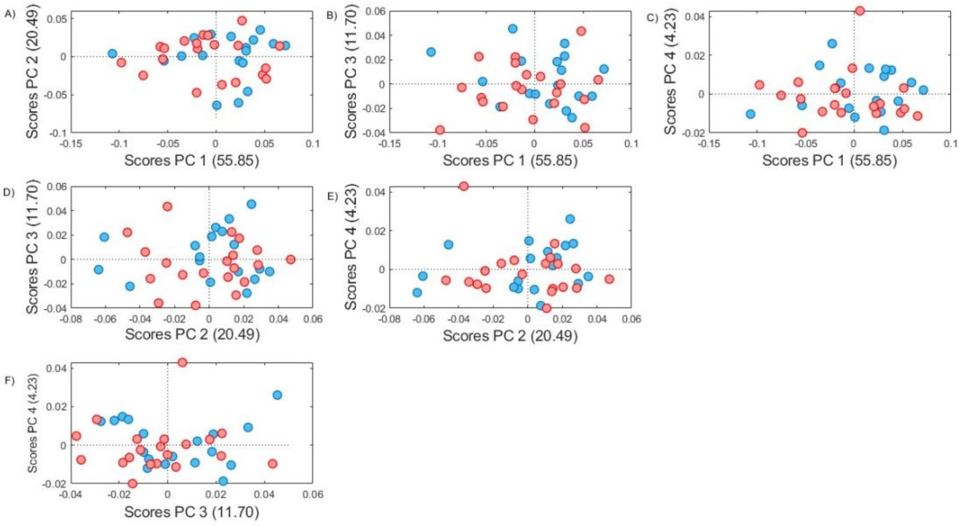

Figure S1. PCA scores plot of a) PC1 *vs.* PC2, b) PC1 *vs*. PC3, c) PC1 *vs*. PC4, d) PC2 *vs*. PC3, e) PC2 *vs*. PC4 and f) PC3 *vs.* PC4 in the total spectra, pre-processed with Multiplicative Scatter Correction. Blue represents the Sham group, while red represents the ovariectomized group.

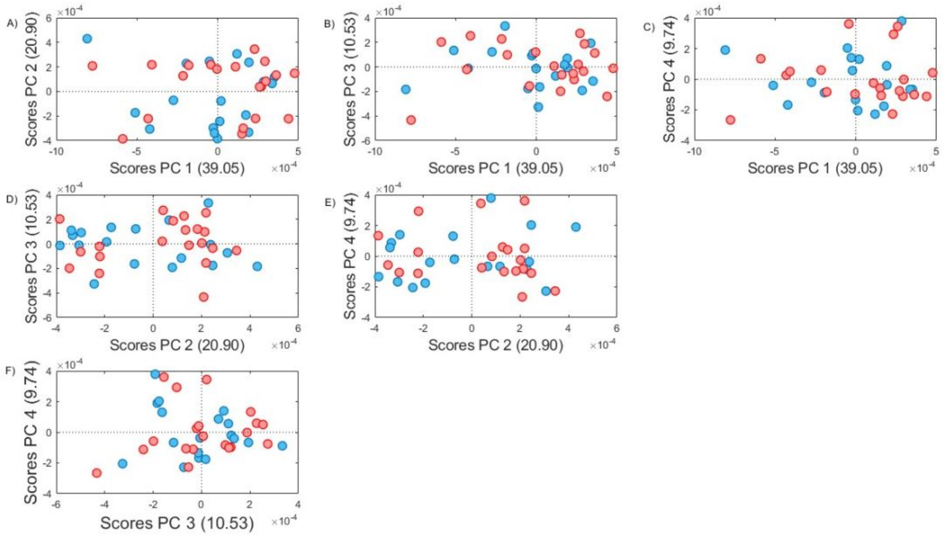

Figure S2. PCA scores plot of a) PC1 *vs.* PC2, b) PC1 *vs*. PC3, c) PC1 *vs*. PC4, d) PC2 *vs*. PC3, e) PC2 *vs*. PC4 and f) PC3 *vs.* PC4 in the total spectra, pre-processed with Multiplicative Scatter Correction and second derivative (15

points). Blue represents the Sham group, while red represents the ovariectomized group.

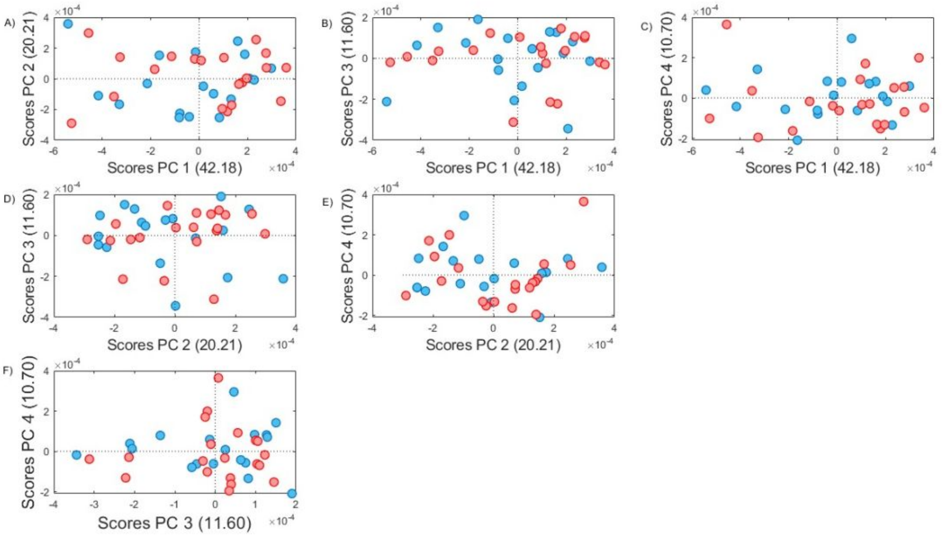

Figure S3. PCA scores plot of a) PC1 *vs.* PC2, b) PC1 *vs*. PC3, c) PC1 *vs*. PC4, d) PC2 *vs*. PC3, e) PC2 *vs*. PC4 and f) PC3 *vs.* PC4 in the total spectra, pre-processed with Multiplicative Scatter Correction and second derivative (21 points). Blue represents the Sham group, while red represents the ovariectomized group.

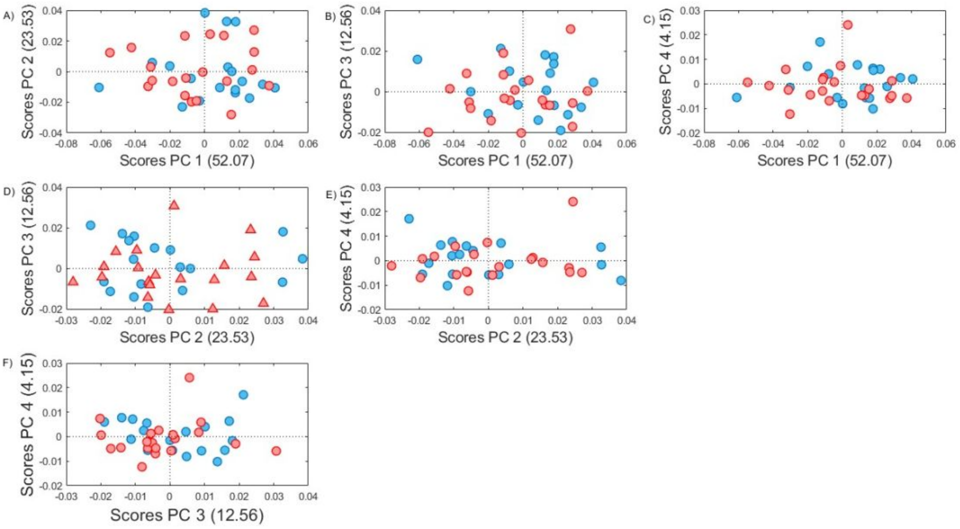

Figure S4. PCA scores plot of a) PC1 *vs.* PC2, b) PC1 *vs*. PC3, c) PC1 *vs*. PC4, d) PC2 *vs*. PC3, e) PC2 *vs*. PC4 and f) PC3 *vs.* PC4 in the total spectra, pre-

processed with normalization. Blue represents the Sham group, while red represents the ovariectomized group.

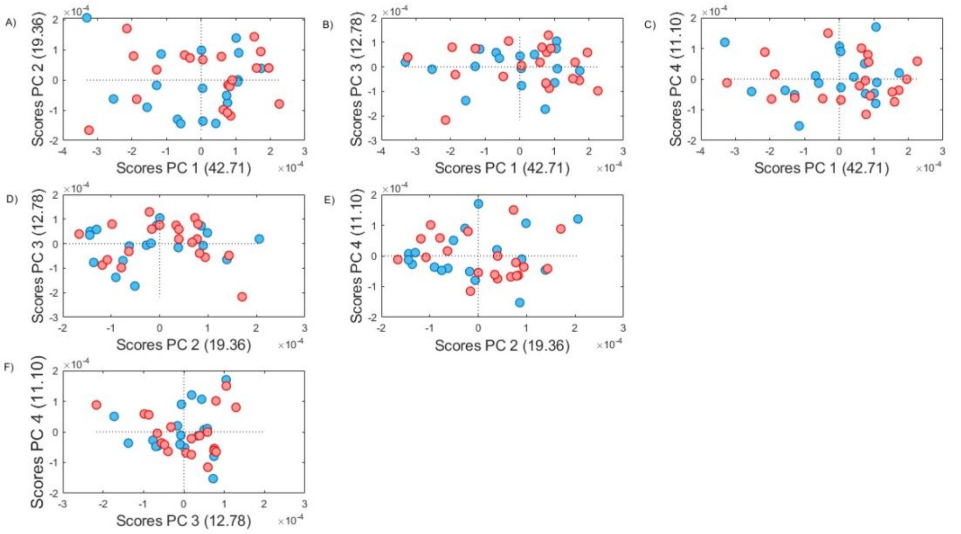

Figure S5. PCA scores plot of a) PC1 *vs.* PC2, b) PC1 *vs*. PC3, c) PC1 *vs*. PC4, d) PC2 *vs*. PC3, e) PC2 *vs*. PC4 and f) PC3 *vs.* PC4 in the total spectra, pre-processed with normalization and second derivative (21 points). Blue represents the Sham group, while red represents the ovariectomized group.

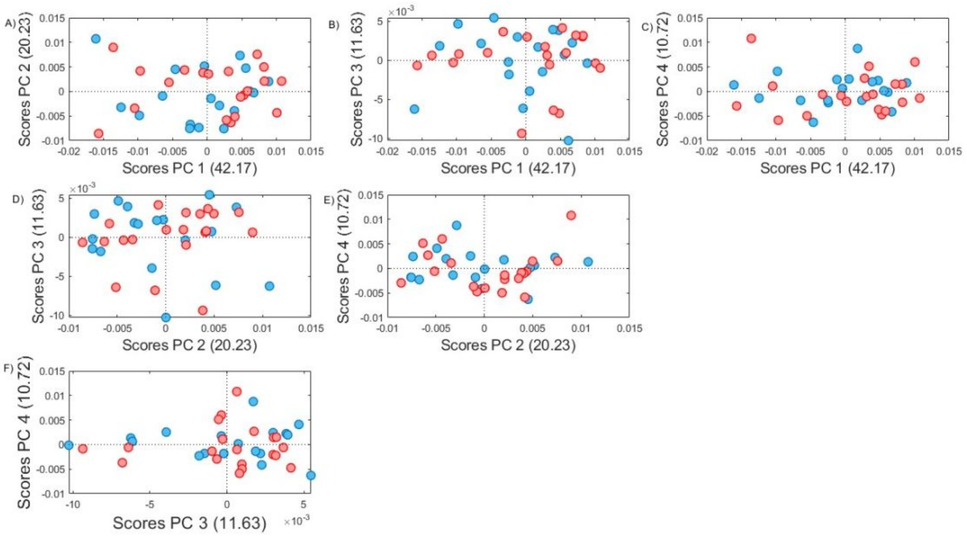

Figure S6. PCA scores plot of a) PC1 *vs.* PC2, b) PC1 *vs*. PC3, c) PC1 *vs*. PC4, d) PC2 *vs*. PC3, e) PC2 *vs*. PC4 and f) PC3 *vs.* PC4 in the total spectra, pre-processed with Standard Normal Variate and second derivative (21 points). Blue represents the Sham group, while red represents the ovariectomized group.

PLS-DA MODEL

Table S1. Performance measures and characteristics of the PLS-DA model of 38 serum samples from ovariectomized and SHAM rats evidenced by ATR-FTIR spectroscopy. Pre-processed with Multiplicative Scatter Correction.

Set	LV	SENS (%)	SPEC (%)	FPR (%)	FNR (%)	F – score	ACC
Training	4	0.76	0.35	0.64	0.23	0.62	0.55
Test	4	0.50	0.62	0.50	0.50	0,76	0.72

LV: Latent variables; SENS: Sensitivity; SPEC: specificity; FPR: False Positive Rate; FNR: False Negative Rate; ACC: Accuracy.

Table S2. Performance measures and characteristics of the PLS-DA model of 38 serum samples from ovariectomized and SHAM rats evidenced by ATR-FTIR spectroscopy. Pre-processed with Multiplicative Scatter Correction and second derivative (19 points).

Set	LV	SENS (%)	SPEC (%)	FPR (%)	FNR (%)	F – score	ACC
Training	4	1.00	0.92	0.00	0.00	1.00	0.96
Test	4	0.80	0.66	0.33	0.20	0,72	0.72

LV: Latent variables; SENS: Sensitivity; SPEC: specificity; FPR: False Positive Rate; FNR: False Negative Rate; ACC: Accuracy.

Table S3. Performance measures and characteristics of the PLS-DA model of 38 serum samples from ovariectomized and SHAM rats evidenced by ATR-FTIR spectroscopy. Pre-processed with normalization.

Set	LV	SENS (%)	SPEC (%)	FPR (%)	FNR (%)	F – score	ACC
Training	4	0.80	0.71	0.28	0.15	0.78	0.77
Test	4	0.80	0.50	0.50	0.20	0,66	0.63

LV: Latent variables; SENS: Sensitivity; SPEC: specificity; FPR: False Positive Rate; FNR: False Negative Rate; ACC: Accuracy.

Table S4. Performance measures and characteristics of the PLS-DA model of 38 serum samples from ovariectomized and SHAM rats evidenced by ATR-FTIR spectroscopy. Pre-processed with second derivative (19 points).

Set	LV	SENS (%)	SPEC (%)	FPR (%)	FNR (%)	F – score	ACC
Training	4	1.00	1.00	0.00	0.00	1.00	1.00
Test	4	0.80	0.50	0.50	0.20	0,66	0.63

LV: Latent variables; SENS: Sensitivity; SPEC: specificity; FPR: False Positive Rate; FNR: False Negative Rate; ACC: Accuracy.

Table S5. Performance measures and characteristics of the PLS-DA model of 38 serum samples from ovariectomized and SHAM rats evidenced by ATR-FTIR spectroscopy. Pre-processed with normalization and second derivative.

Set	LV	SENS (%)	SPEC (%)	FPR (%)	FNR (%)	F – score	ACC
Training	4	1.00	1.00	0.00	0.00	1.00	1.00
Test	4	0.80	0.50	0.50	0.20	0,66	0.63

LV: Latent variables; SENS: Sensitivity; SPEC: specificity; FPR: False Positive Rate; FNR: False Negative Rate; ACC: Accuracy.

References

(1) Peris-Díaz, M. D.; Krężel, A. A Guide to Good Practice in Chemometric Methods for Vibrational Spectroscopy, Electrochemistry, and Hyphenated Mass Spectrometry. *Trends Anal. Chem.* 2021, *135*, 116157. https://doi.org/10.1016/j.trac.2020.116157.

(2) Bro, R.; Smilde, A. K. Principal Component Analysis. *Anal. Methods* 2014, *6* (8), 2812–2831. https://doi.org/10.1039/c3ay41907j.

(3) Brereton, R. G.; Lloyd, G. R. Partial Least Squares Discriminant Analysis: Taking the Magic Away. J. Chemometrics 2014, 28 (4), 213–225. https://doi.org/10.1002/cem.2609.

(4) Ballabio, D.; Consonni, V. Classification Tools in Chemistry. Part 1: Linear Models. PLS-DA. Anal. Methods 2013, 5 (16), 3790–3798. https://doi.org/10.1039/c3ay40582f.
